# Disparities in Methods Used to Determine Microplastics in the Aquatic Environment: A Review of Legislation, Sampling Process and Instrumental Analysis

**DOI:** 10.3390/ijerph18147608

**Published:** 2021-07-17

**Authors:** Jan Halfar, Kateřina Brožová, Kristina Čabanová, Silvie Heviánková, Alena Kašpárková, Eva Olšovská

**Affiliations:** 1 Faculty of Mining and Geology, VŠB-Technical University of Ostrava, 17. listopadu 15/2172, 708 00 Ostrava, Czech Republic; katerina.brozova.st1@vsb.cz (K.B.); kristina.cabanova@vsb.cz (K.Č.); silvie.heviankova@vsb.cz (S.H.); alena.kasparkova@vsb.cz (A.K.); 2 Centre for Advanced and Innovative Technologies, VŠB-Technical University of Ostrava, 17. listopadu 15/2172, 708 00 Ostrava, Czech Republic; eva.olsovska@vsb.cz; 3 Nanotechnology Centre, CEET, VŠB–Technical University of Ostrava, 17. listopadu 15/2172, 708 00 Ostrava, Czech Republic

**Keywords:** microplastics, aquatic environment, legislation, determination, water

## Abstract

Plastic particles smaller than 5 mm, i.e., microplastics, have been detected in a number of environments. The number of studies on microplastics in marine environments, fresh water, wastewater, the atmosphere, and the human body are increasing along with a rise in the amounts of plastic materials introduced into the environment every year, all contributing to a range of health and environmental issues. Although the use of primary microplastics has been gradually reduced by recent legislation in many countries, new knowledge and data on these problems are needed to understand the overall lifecycle of secondary microplastics in particular. The aim of this review is to provide unified information on the pathways of microplastics into the environment, their degradation, and related legislation, with a special focus on the methods of their sampling, determination, and instrumental analysis. To deal with the health and environmental issues associated with the abundance of microplastics in the environment, researchers should focus on agreeing on a uniform methodology to determine the gravity of the problem through obtaining comparable data, thus leading to new and stricter legislation enforcing more sustainable plastic production and recycling, and hopefully contributing to reversing the trend of high amounts of microplastics worldwide.

## 1. Introduction

Recently, research in microplastics has proliferated due to a number of reasons associated with the use of plastics, in general, which have long been part of our daily lives. Besides the benefits of plastics and microplastics, they have also become pollutants, contaminating all the constituents of the environment. Plastic waste has lately become one of the most serious environmental issues, as it has low biodegradability and is managed inappropriately [1]. According to the latest data from 2019, the worldwide production of plastics amounted to 370 million tons, of which 58 million tons are reported for Europe alone [2].

Microplastics are mostly defined as particles smaller than 5 mm [3] and come in different shapes such as fibers, pellets, fragments, flakes, and beads [4]. Primary microplastics are produced for their abrasive properties, mainly as consumer goods in the cosmetics industry or cleaning agents. On the other hand, secondary microplastics are formed by the gradual degradation and subsequent fragmentation of larger plastic particles and are considered the predominant source of microplastic pollution in the environment [5,6,7].

Microplastics have been discovered almost everywhere: in the ocean [8], fresh water [9], wastewater [10], coastal areas [9,11], soil [12], sediment [13], atmosphere [14], various organisms with different frequencies, feeding patterns and trophic levels [15,16] and in food and agricultural systems [17]. The abundance of these particles may have a potential impact on human health [7].

The methods most commonly used for microplastics’ identification and quantification are infrared spectroscopy with Fourier transform (FTIR) and Raman spectroscopy [18,19]. Other methods used to detect microplastics in various environments include gas chromatography in conjunction with a mass spectrometry (GC/MS) detector, thermal method (TGA) scanning electron microscopy in conjunction with energy dispersive X-ray spectroscopy (SEM EDX), or X-ray fluorescence (XRF) [19,20,21,22].

In a number of studies that deal with the analysis of microplastics, we can find different procedures for sample preparation and different methodologies in the analysis of microplastics themselves. However, this can cause problems in obtaining valid results [19,23,24]. Related to this are complications in the preparation of comprehensive legislation, which would be based on clear procedures for the preparation, identification and evaluation of microplastics in the environment and would include primary and secondary microplastics. Currently available legislation focuses mainly on microplastics in cosmetics and therefore lacks the constraints necessary to achieve sustainable restrictions on the use of plastic products [12,25,26].

The aim of this review is to:

- Provide the reader with information on plastics and microplastics and their occurrence in the environment, specifically in sediments and soil, sea and ocean, fresh water and groundwater (Section 2);
- Present an overview of currently used methods of the determination of microplastics in water, including the details on sampling and sample processing (Section 3);
- Review the subsequent instrumental analyses of microplastics in water with a focus on the two most used methodologies, i.e., Raman spectroscopy and infrared spectroscopy with Fourier transformation (Section 4);
- Provide a critical overview of the legislation on microplastics, namely in the USA, European Union, China, Argentina, UK and Canada (Section 5).

According to these mentioned sections, this review presents information that demonstrates the complexity of formulating and preparing legislation in the field of microplastics. The information from the studies shows considerable differences not only in the presentation of the results, but mainly in the description of the methods used. For this reason, current and forthcoming legislation focuses mainly on restricting disposable plastic materials (such as disposable packaging, microbeads in cosmetics and detergents, and plastic straws), which is important, but little control over its effectiveness is possible. Only if the methodology is uniform, it will be possible to propose laws that cover a much wider range of possible restrictions on microplastic pollution in the environment (such as compulsory recycling, the production of new plastic products from recycled material, and energy recovery from non-recyclable plastic waste) and their effectiveness can be monitored.

## 2. Plastics and Microplastics

**Figure 1:**
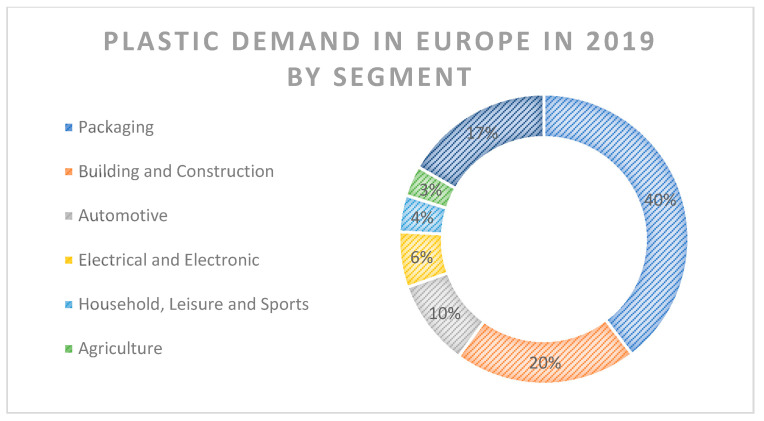
Plastic demand in Europe in 2019 by segment (Data: Plastic Europe 2020).

Plastic materials have been used in a wide range of industries due to their versatility and have become indispensable parts of modern life. According to Plastic Europe data, in 2019, 368 million tons of plastics were produced worldwide, out of which 16% are attributed to Europe. Asia holds the lead in the production of plastics with 51% of global production in 2019 [2]. Figure 1 shows the plastic demand in Europe in 2019 by each segment, where packaging dominates with 40% of total demand (Figure 1).

Due to the continuously growing interest in plastics, an identical growth trend in their production may be expected in the upcoming years. Almost half of the produced plastics are disposable plastic products, which often turn into waste without further use. It is estimated that up to 8 million tons of plastics end up in the oceans each year [27]. As a result, there are many polymer compounds in circulation these days, the most famous of which are: polypropylene (PP), polyethylene (PE) and its modifications such as low-density polyethylene (PE-LD), linear low density polyethylene (PE-LLD), high density polyethylene (PE-HD), medium density polyethylene (PE-MD), polyvinyl chloride (PVC), polyurethane (PUR), polyethylene terephthalate (PET), nylon (NY), polyester (PES), and polystyrene (PS) [2,4,28,29,30].

### 2.1. The Pathways of Microplastics into the Environment

Microplastics entering the environment are either produced directly during the manufacturing process (primary MPs) or form by the degradation and fragmentation of plastic waste (secondary MPs) [31,32,33,34,35].

Microbeads found in personal care products, microfibers from textile products and resin pellets used to create other plastic products can be classified as microplastics. They are also produced specifically for their abrasive characteristics [5,7,30].

As microplastics are also suitable for cleaning and exfoliating skin or teeth, they are very often used in the cosmetics industry. They can be found in personal care cosmetics and cleaning products such as shower gels, toothpaste, liquid soap, hair care, masks, scrubs, facial cleaners, nail care and decorations, glitter, make-up, lip care, deodorants, sun care, etc. These kinds of products include polyethylene (PE), polyester (PES), polyvinyl chloride (PVC) and high density polyethylene (HDPE). Microplastics are found both in rinse-off cosmetics and leave-on cosmetics. Rinse-off types enter the environment immediately after use, most often through wastewater ending up in the wastewater treatment plant. Standard wastewater treatment processes mainly retain larger fragments of microplastics and absorb them into sewage sludge; however, many that are not retained always enter the aquatic environment. It should be noted that the sewage sludge is largely deposited on agricultural land, which means direct contamination of the soil with the microplastics. Leave-on types can also be washed after a certain exposure time and thus end up in an aquatic environment (in the same way as rinse-off types) or they can be removed from the skin with a napkin or a cotton pad, which usually ends up as waste in landfills [5,7,30].

Another major source of primary microplastics in the environment is the textile industry. Production and trading activities of textile industries pollute the environment with synthetic fibers composed of polyester, acrylic, polypropylene, polyethylene and poly-amide. Their presence in the environment is largely attributed to the wastewater generated during the production, coloring and washing of textiles (both industrial and domestic), with the wastewater ending up in the wastewater treatment plant and causing the same problem as in the cosmetics industry. Mechanical stress also acts during the production process, thus releasing synthetic fibers. The drying process, packaging and transport, in which many microplastics are released, lead to further environmental pollution [5,6,7].

Other important primary MPs are pellets (also known as nurdles) produced in the recycling of plastics Although they are produced with the environmental measures in mind, these pellets enter the environment during their production, transport and subsequent processing, where they act as pollutants. Resin pellets are used as a raw material for the further production of plastics in the personal care industry, automotive, agriculture, construction, and sporting goods industries and the packaging industry [36].

Secondary microplastics, on the other hand, are most likely to result from the gradual breakdown of larger plastic pieces that occur mainly in nature and water as plastic wastes. These are most often disposable plastic packaging and plastic household utensils, and the most common waste is specifically: plastic bags, bottles, straws, cups, storage containers, ropes, caps, cool boxes, floats, polystyrene utensils, pipes, containers, fishing nets, glass fiber textiles, cigarette filters, etc. Another source may be vehicle tires (sometimes classified as primary microplastics), which are abraded while driving [6,37]. The process of secondary MPs’ formation and their occurrence in the aquatic environment is attributed to the action of wave action, wind abrasion, UV radiation, hydrolysis and bioassimilation by microorganisms. UV radiation weakens and degrades the plastic structure, which subsequently breaks down into smaller particles as a result of mechanical wear [38,39]. Another possible reason for the disintegration of plastic products into smaller parts may be the deformation and stress points caused by production [40].

### 2.2. Occurrence of Microplastics in the Environment

**Figure 2:**
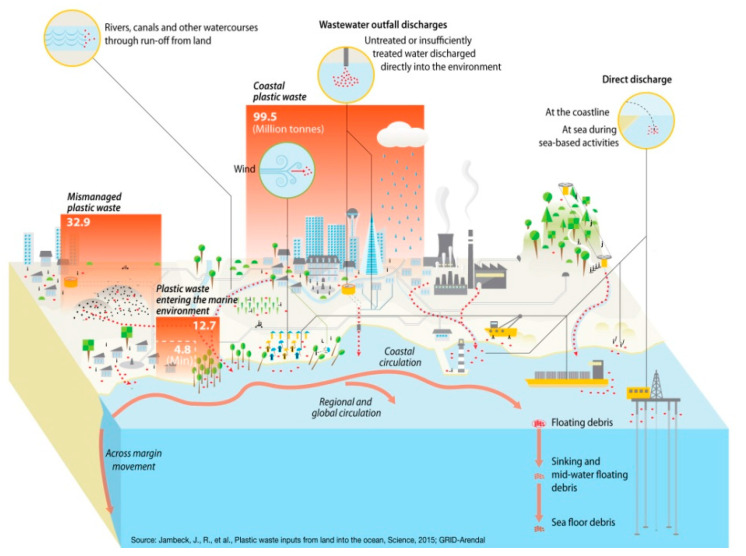
The microplastic sources and pathways into the environment. Image available online at https://www.grida.no/resources/6922 (accessed on 11 June 2021). Author credit: Maphoto/Riccardo Pravettoni.

The fact that microplastics have already become part of the environment is supported by a number of studies. For this reason, in recent years, the attention of the scientific community has increasingly focused on the occurrence of MP in the environment.The main research directions include lakes, rivers, sediments, oceans and seas, soil, and the atmosphere. Modern human life involves extensive applications of plastics, which, when used, often pollute the environment and have negative impacts on all components of the food chain. As shown in Figure 2 above, the distribution of microplastics in the environment probably comes mostly from plastic waste and its wear debris.

The following sections review the occurrence of microplastics in different water-related environments (Sediments and Soil; Sea and Ocean; Fresh Water and Groundwater).

#### 2.2.1. Sediments and Soil

Most plastic waste ends up either in the aquatic environment or on land. On land, these microplastics are deposited in soil or sediments, the sources of which are extensive. Increased concentrations of microplastics can be found, for example, near industrial zones, ports, and cities [12]. Some MPs enter the soil in connection with sludge management in wastewater treatment plants. From there, the treated sewage sludge may be transported onto agricultural land due to its fertilizing effects. However, this treated sewage sludge still contains significant amounts of MPs that pass into agricultural land [41,42,43]. Corradini et al. (2019) carried out research on 31 fields where sewage sludge was used and it was found that with increasing applications of sewage sludge, the content of microplastics in the soil increased non-linearly [44]. Researchers also focused on the transport of microplastics in soil through terrestrial organisms such as earthworms or springtails [23].

Another area of interest is sediment containing a wide range of substances settled from water and deposited in the landscapes of rivers, lakes, or estuaries. It has been widely established that sediment is the final reservoir for MPs [45]. The reason is an increase in biofilm on the surface of the plastic particle, or adherence to the excrements of animals, which causes weight gain leading to sedimentation [40]. In rivers, MPs are carried by water to the river estuaries, or into areas with negligible flow, where they fall to the bottom and settle as sediment. In the estuaries, the situation is more complex and depends on several factors. An important factor is the flow at the delta, which determines whether MPs travel further into the ocean or settle on the coast. In the case of the sedimentation of MPs, accumulation and ecological risks occur not only for benthic communities [40,46]. The problem of bioaccumulation in organisms is an increasingly discussed topic because organisms search for food in sediments. This way, plastic particles get into different organisms and travel further down the food chain.

Wang et al. (2019) conducted research in the Southern Yellow Sea, where they found that MPs’ concentrations were up to 100 times higher in the upper sediment layer (0–5 cm) than in the deeper sediment layers [46]. Similar research was conducted by Antunes et al. (2018), who collected and analyzed a total of 162 sediment samples from the Portuguese coast. Samples were always taken on the beach near a major site such as a port, industrial center, or estuary. In all samples, microplastics were confirmed, and the highest measured concentration was 1964 + −3621 items per square meter [47].

Corradini et al. (2021) conducted research on the occurrence of microplastics in various soils in the central valley of Chile, where urbanization, agricultural, and mining operations are typical. A total of 240 samples of soils from different types of natural grasslands, rangelands, croplands, and pastures were identified. A total of 43% of the samples contained microplastics, and it was found that the occurrence of microplastics is conditioned by land use. The most frequent microplastic contamination was found in croplands (57%) and pastures (44%). In contrast, in natural grasslands (20%) and rangelands dominated by shrubs (3%), microplastic pollution was less frequent [48].

#### 2.2.2. Sea and Ocean

Research on microplastics in the marine environment dates back to the 1970s, when in 1972 Carpenter and Smith Jr. published the first study on the content of plastics in the Sargasso Sea [49]. Since then, the number of publications on this topic has been increasing exponentially. As of 27.04.2021, the search using keywords “microplastic AND marine” in the Web of Science database produced 1971 results. The growing interest in this topic proves its importance in the scholarly community, and it shows that the disproportionate pressure of humankind poses a serious threat to the marine ecosystem [50].

The introduction of microplastics into the marine environment used to be attributed to shipping, cargo handling, shipping accidents, and targeted discharge of plastic waste. Today, however, many microplastics come from rivers that flow into the sea [27,51]. Based on the current data from the International Union for Conservation of Nature, approximately 8 million tons of plastic waste reach the seas and oceans every year, which is unsustainable when aiming to maintain good water quality [52].

The undisputed content of MPs in the marine environment is a major problem for future development and a threat not only to the ecosystems, but also to humankind. Nevertheless, only a small number of studies so far confirm that microplastic particles pose a threat to marine biota [50,53,54,55]. MPs accumulate not only in the aquatic environment, but also in organisms, which is becoming a matter of great concern.

The accumulation of microplastics in marine ecosystems has a growing tendency worldwide. The most affected areas are river deltas, coastal areas with dense settlement and industry, and subtropical oceanic gyres [13]. Microplastic particles are consumed very often by a group of aquatic animals, since the particle size is equivalent to the size of plankton [50]. The occurrence of fibers and grains has also been confirmed in marine sediments, causing, for example, the uptake of these microplastic fragments by a group of benthic animals [56]. The transfer of microplastic particles between trophic species has not yet been confirmed with certainty. However, studies confirming the transfer of these fragments in the food chain already exist. Food chain transmission also increases the risk of accumulation in higher trophic species.

Huang et al. (2021) demonstrated the toxic impact of microplastics on mussels (*Mytilus coruscus*) at environmentally relevant concentrations. Mussels were exposed to three certain concentrations of microplastics in water tanks with clean filtered seawater for fourteen days. The presence of microplastics in the digestive tract of mussels was detected. In addition, physical damage such as scratches, perforations, and gastrointestinal obstruction has occurred [57].

Wang et al. (2021) conducted research on the microplastic impact and accumulation in a predatory marine crab (*Charybdis japonica*). Crabs were exposed to microplastics for one week (particle size: 5 μm). The presence of microspheres in the hepatopancreas, guts, gills, and also muscles was demonstrated (from the highest to the lowest concentration). Once the concentration of microplastics in the hepatopancreas exceeded 3 mg/g, the damage of the crab liver and neural activity occurred [58].

#### 2.2.3. Fresh Water and Groundwater

Fresh water and groundwater together with atmospheric precipitation are sources of water on land. For this reason, it is important to thoroughly observe fresh water and groundwater for the presence of microplastics. Plastic waste settled in river basins is often a source of pollution in the rivers and lakes to which plastic waste is transported and can be accumulated [31,59]. Most studies focus on microplastics in the oceans, but it is equally important to investigate inland water too, where the content of MPs is likely to be similar.

The content of microplastics in fresh waters has been verified in previous studies,, including the rivers Danube [60], Elbe, Mosel, Neckar and Rhine in Germany [61], Vembanad Lake in India [62], Dongting Lake and Hong Lake in China [63] or the Chishui River in China [59]. MPs in fresh water and groundwater have their sources on land. From there, through atmospheric precipitation and dry deposition, microplastics enter water bodies, where MPs can be accumulated in sediments or are carried to the seas and oceans [9].

Xiong et al. (2018) examined the content of microplastics in the largest inland lake, Qinghai, recently becoming a popular tourist destination. Microplastic pollution was expected and sampling occurred in 2016 using trawls. Samples of fish were also collected and analyzed. The presence of microplastics was confirmed in all cases. The results were interesting because there is no industrial center near the study area and the population density is also very low. However, in recent years, the number of tourists has risen to 1 million. Therefore, increasing tourism can be considered negative in terms of microplastic content too [64].

Further research was conducted by Campanale et al. (2019) in the Italian river Ofanto. In this case, the samples were taken by trawl plankton nets, and their subsequent processing and analysis was made by microscope and pyrolysis–gas chromatography/mass spectrometry (Py–GC/MS). The authors reported a total of five samples from February 2017 to May 2018. In all investigated samples, the presence of microplastics was confirmed to a degree comparable to or greater than in other studies [65].

Another investigated group of waters is wastewater. Due to the formation of primary microplastics, it is very likely that wastewater flowing into wastewater treatment plants (WWTPs) will be heavily burdened with these MPs. Some studies even identified a wastewater treatment plant to be a source of MPs in relation to the river, while others have refuted this claim [6,66,67]. As for this topic, the point of interest is to clarify the mechanisms of MPs’ passage via WWTPs, the efficiency of disposal, and the quantity of microplastics discharged further into the recipient.

Hidayaturrahman and Lee (2019) investigated the possibility of removing MPs at three wastewater treatment plants fitted with different technologies at the tertiary stage of the treatment system. They used the following methods: ozonation, a membrane filtration, a disc filter and a quick sand filtration.. Analyzed samples were taken at five sites for each type of technology, specifically, at the inlet, downstream of the primary purification stage, downstream of the secondary purification stage, after coagulation at the tertiary stage, and lastly at the outlet to the recipient. The authors of this study used a microscopic imaging technique to analyze the microplastic content and do not specifically describe any technique that would confirm the composition of the observed materials [68].

Zhang et al. (2021) conducted research on four wastewater treatment plants with similar treatment technology in Guilin City. Microplastics were removed firstly at the pretreatment stage where large pieces of solid plastic particles were separated. It was shown that 40–50% of microplastics were removed from the pretreatment stage. This was followed by a secondary stage in which microplastics are removed by biological mechanisms. At the outflow from the WWTP, it was found that the rate of removed microplastics was around 90–94% [67].

However, it is clear from the results of the study that the difference between the microplastic inlet and outlet concentrations is very noticeable [67]. Each process has the potential to remove these pollutants from wastewater. However, the subsequent tertiary treatment system is able to remove up to 99% of MPs on average. Of all tertiary treatment methods, ozone technology has performed best. Here, the MPs’ concentration was reduced by 99.2%. This was followed by a membrane disc filter with 99.1% efficiency and the last one was a sand filter with an efficiency of 98.9%. This research shows that ozone technology is a promising way to remove MPs at a WWTP [6,68].

Although groundwater is a less risky area in terms of possible contamination than fresh water, an insufficient number of studies also concern the likely content of microplastics in groundwater [69]. The presence of microplastics in groundwater can be caused by the deposition of contaminated organic matter on the soil. However, this can be associated with the deposition of sewage sludge, which contains microplastics, on arable land, from where these particles are subsequently transported vertically to the lower solid layers [41].

## 3. Methods of Determination of Microplastics in Water

Studies on the topic of microplastics in waters, sediments, and biota have long been confronted with inconsistent methodologies for sampling, sample preparation, and processing with subsequent evaluation. This causes a considerable difficulty in comparing results across studies. Very often, two studies in a similar environment show quite different results because of completely different methods. The following section describes the most common methods used to retrieve MPs.

### 3.1. Sampling

Reducing the sample volume and bulk sampling are the two main categories of the methods used in sample collection. The bulk sampling technique is suitable for more accurate knowledge about fragments and fibers in samples. In this case, samples are drawn into glass or metal bottles. In bulk sampling, it is also possible to use suction pumps or sample bottles, such as a Niskin bottle [70,71]. The volume-reduced sampling procedure is commonly used to collect particles from large water volumes [72]. Trawls are often used for the sampling of fresh water, which are placed at different depths and pulled at different speeds. For example, neuston nets, manta trawls, and plankton nets are widely used (Table 1).

Kor and Mehdinia (2020) used a trawl net with a mesh size of 300 µm and a rectangular hole of 130 × 30 cm for sampling in the Persian Gulf. Here, the GPS coordinates at the beginning and end of the sampling were used to calculate the sampled area. To reduce wind and wave exposure, the net was mounted on the windward side of the ship at a speed of 2–3 knots [73].

Schönlau et al. (2020) drew samples in the Baltic Sea region using two sampling methods. Manta trawl was the first method used for the collection of 24 samples (12 locations with 2 samples at each). The second one was an in situ filtering pump used for 11 samples. After sampling, the content of the manta trawl with 333 µm mesh size was transferred into a metal filter with 300 µm mesh size and, after that, samples were stored in glass jars. The filtering pump was fitted with three metal filters with mesh sizes of 50, 300, and 500 µm and they were removed after sampling and stored in metal jars covered by aluminum foil. A higher amount of microplastic particles was found using the manta trawl method in the Western Gotland Basin [71].

Cai et al. (2018) used two sampling methods for sampling in the South China Sea. The first was a Bongo-type trawl with a 333 µm mesh size towed behind a vessel. Upon withdrawal, the contents were washed into a glass container with Milli-Q water and stabilized by the addition of 30% formalin. The second type of sampling was the use of a centrifugal pump supplemented with water meters and a PVC hose. The water was immediately filtered through square filters of 5 mm, 154 µm, and 44 µm mesh sizes. A 5-mm fraction was excluded from observation. Fractions of 154 µm and 44 µm were coated with aluminum foil and stored at 4 °C [74].

### 3.2. Sample Preparation

For a proper determination of microplastics in water, sample preparation is needed. Methods without further preparation could only be used in pure water without the presence of organic material. In this case, it is necessary to separate the microplastics from the sample matrix. According to the type of water samples, a variety of methods are used, including density separation, filtration, sieving, and chemical digestion of organic matter [100,101,102,103,104]. As Table 1 shows, different methods are used for sample preparation and this causes problems with replicability.

#### 3.2.1. Density Separation

The separation of microplastics from the sample matrix works on the principle of their different densities and it is the most widely used method for sediment samples (Table 1). As the density of commonly used plastic materials ranges from 0.91 g/cm^3^ (polypropylene) to 1.45 g/cm^3^ (polyvinyl chloride) [105], sodium chloride (NaCl), zinc chloride (ZnCl_2_), sodium iodide (NaI), and calcium chloride (CaCl_2_) are the most used substances in density separation. Due to the lowest price, low toxicity and easy preparation, the solution of sodium chloride (NaCl) is mainly used (Table 1). As for other substances, the cost and consequent toxicity of the solutions should be considered before use. After the solution is added, the lower density polymer particles float to the top of the solution due to shaking and settling [12,106,107].

#### 3.2.2. Filtration

Filtration is a process of separating a solid phase from a liquid phase using a filter baffle, when the target mesh size is important. This method could be used in different steps of sample processing, after density separation or chemical digestion and at the beginning of the sampling process. For the laboratory analysis, conventional filtration methods are used, for example, filtration under vacuum or membrane filtration. However, for these methods, glass-fiber, stainless-steel, or membrane filters are used [33,108,109]. All of these filters have their advantages and disadvantages but, to date, there has been no precise methodology for the use of a uniform filter material. Similarly, the mesh size of the filter varies widely from hundreds [110] to tenths of micrometers [111]. The use of different filters and filtration methods can be seen in Table 1.

#### 3.2.3. Chemical Digestion

The abundance of organic matter in the sample is a considerable problem when waters from surface sources (rivers, lakes, oceans) as well as from sediments are examined. Therefore, the organic matter must be removed from the sample, most often by digestion. The digestion of organic matter by using acidic aqueous solutions or oxidizing agents that degrade organic matter structures is most widely used today. However, the risks of damaging microplastic particles or changing their properties are high using some solutions. This may further cause problems in determining MPs. The main solution for the chemical digestion of sediments is hydrogen peroxide H_2_O_2_ (30% or 50%) (Table 1) [109]. In other cases, for example, for the digestion of biota samples potassium hydroxide KOH (10%) and concentrated hot nitric acid HNO_3_ or combinations thereof are used [108,111].

## 4. Instrumental Analysis of Microplastics

Instrumental analysis on particles that are potentially made from plastic is performed to confirm their composition. As we can see in Table 2, the most common methods of instrumental analysis include infrared spectroscopy with Fourier transform (FTIR), Raman spectroscopy (RS), or scanning electron microscopy (SEM). It is also possible to use Pyrolysis-gas chromatography/mass spectrometry (Pyrolysis-GC/MS), thermogravimetry with differential scanning calorimetry (TGA–DSC), liquid chromatography with mass spectroscopy (LC/MS) or energy dispersive X-ray spectroscopy (EDS).

### 4.1. Raman Spectroscopy

The principle of Raman spectroscopy is based on the irradiation of the sample with monochromatic radiation and subsequent reaction of the sample molecules. We observe the Raman scattering of the altered wavenumber, where the lines of the Raman spectrum correspond to the characteristic vibrations of the molecules. By means of Raman spectroscopy, it is possible to examine microplastics in various samples, for example, in the atmosphere, fog, wastewater, sediments, biota, surface water, and groundwater. As reported by Xu et al. (2019), due to the different wavelength settings of the monochromatic radiation source and the different exposure times of the sample, slightly different spectra were obtained for the same plastic types. It was also stated that the measurement of the MPs still lacks the unification of the substrate on which they are captured. Various substrates, such as glass fiber filters, silicone filters, etc., have been reported in studies [35].

Cho et al. (2021) made a feasibility study for online polyethylene (PE) particle detection in water by using Raman spectroscopy. They used perfluorohexane (PFH) as a medium for capturing polyethylene particles dispersed in water. PFH was added to the water in a special L-shape tube where it formed droplets. Dispersed polyethylene particles were captured on surface of PFH droplets due their high density and hydrophobicity. Whole droplets with PE on their surface were sampled using the wild area illumination (WAI) scheme. The authors recovered 95.9% of the PE particles from water and tried to adapt this method for other types of plastics [112].

### 4.2. Infrared Spectroscopy with Fourier Transformation

Fourier transform infrared spectroscopy is one of the most widespread instrumental methods for determining the material of which microplastics are composed. In principle, it is the action of monochromatic radiation that acts on a given substance. By means of the radiation absorbed or reflected further by the Fourier transform, we obtain a vibration spectrum. Measurements can also be made of a crystal (FTIR-ATR) supplementing the common FTIR instruments. However, by using the vibrational spectrum, it is possible to determine typical peaks for a given structure of substances. After that, the spectrum is compared with the spectral library, which could be internal or external.

Comparing spectra with different libraries causes different results, where the Omnic Spectral Library, the Nicodom Polymers Library, and the Shimadzu Materials Library are the mainly used libraries [35]. However, sometimes only a slight deviation can be substantial and comparisons of results between studies are thus useless. The consistent use of these libraries could also contribute to the unification of measurement results.

Moreover, Minteng et al. (2019) used Micro-FTIR analysis to determine the content of microplastics in drinking water from underground sources. Ten out of 24 samples of raw and drinking water were confirmed for positive content of microplastics. PEST, PVC, PE, PA, and epoxy resin materials were confirmed by Micro-FTIR analysis [69].

Corani et al. (2020) used Micro-FTIR Nicolete iN10 to measure microplastic particles smaller than 100 µm in gills and hepatopancreas from Pacific oysters, which were sampled in Canal Pordelio, Italy. Each sample was analyzed by Micro-FTIR in transmittance mode with a spectral range of 4000–1200 cm^−1^. All spectra were identified by comparing these with libraries and only the spectrum with a match higher than 65% was accepted. The authors state that the total amount of microplastics was 329.849 ± 1149 in gills and 238.931 ± 677 in hepatopancreas [113].

## 5. Microplastics in Legislation

**Figure 3:**
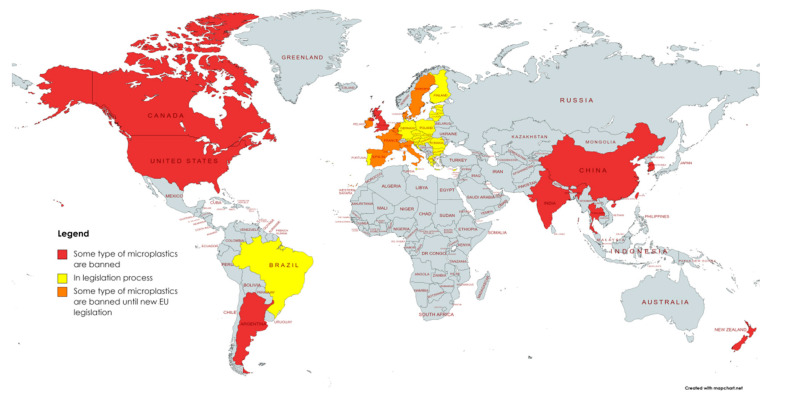
Countries enforcing legislation on primary microplastics (created with mapchart.net©, 2021).

With the growing awareness and interest in a detailed examination of the abundance of microplastics in the aquatic environment, legislation is also being drafted. This legislation should make it possible to reduce the amount of plastic particles in the aquatic environment. If this problem is not adequately addressed at the legislative level, steps will not be taken to reduce the amounts of microplastics already present in waters. The following section briefly describes the current legislation of the United States of America, the European Union, China, and some other countries. The creation of new legislation may be impeded or at least complicated by the problematic fragmentation of the methodology (Table 1) used in the determination of microplastics, which is very important for its preparation. However, some countries (Figure 3) have already started implementing their legislation, but it is mainly focused on rinse-off cosmetics and personal care products only, i.e., the sources of primary microplastics.

### 5.1. Legislation in the USA

California, New York, and Illinois are among the states that have spoken out against the use of primary microplastics. These states have launched a fight against the use of these substances at local and subsequent state level. Subsequently, Frank Pallone Jr., Representative of Congress from the State of New Jersey, introduced a proposal to ban the use of plastic microparticles in 2015. As a law, it was unanimously passed in Congress and was signed by President Barack Obama on 28 December 2015. In short, the H.R.1321-Microbead-Free Waters Act of 2015 prohibits the production of rinsing cosmetics with the deliberate addition of plastic microparticles and their subsequent sale from 1 January 2018 [114].

However, the law is also criticized for its limited scope and many loopholes. There is also little support for the development of biodegradable alternatives to banned microparticles [115]. In addition to this law, there are also a number of laws for the protection of waters and oceans and against their pollution. Although these are the first steps in the fight against microplastics in the aquatic environment, they have sufficient scope and have been an inspiration for drafting legislative documents in other countries.

### 5.2. Legislation in the European Union

Negotiations are under way in the European Union on the extension of Annex XVII to REACH. ECHA (European Chemicals Agency) proposes a sharp reduction in the use of targeted microplastics in a wide range of sectors. Proposed restrictions are listed in the Annex XV Restriction Report—Intentionally added microplastics, which is available online on ECHA’s website. This is a far greater restriction on the use of microplastics than in previous US legislation. Such widespread restrictions on the use of targeted microplastics in products mean that the legislative authorities already have sufficient information on this issue. Consequently, they also know the risks that can become a reality if they are not solved.

Expert opinions are currently being drawn up, and 2022 is set as a realistic year for the approval and entry into force of the extension of Annex XVII. In 20 years, microplastic emissions are likely to be reduced by 85%, saving an estimated 400,000 metrictons of released microplastics [116,117].

However, some EU member states have already released their legislation in the field of microplastics, for example, Denmark, Sweden, and Belgium [118]. The Swedish government banned cosmetic products with added microplastics such as toothpastes, shampoos, or shower gels. The ban came into force on 1 July 2018, but stock could be sold in shops until 1 July 2019 [119]. Similar proposals were presented by the Danish government, which tried to temporarily ban rinse-off cosmetic products with added microplastics. The available law draft prohibits the use of microplastics smaller than 5 mm in rinse-off cosmetics [120]. Rinse-off cosmetics with microplastics are banned in member states of the European Union such as Spain, France, Italy and Ireland [121]. However, these states are still waiting to unify their legislation with the European Union, which may last for the next few years.

### 5.3. Legislation in China

China, as one of the largest manufacturers of plastics and cosmetics, is also developing new legislation to prevent the use of targeted microplastics in the cosmetics industry. Information on this prohibition was given in the Catalog of Guidelines for the Adaptation of Industrial Structures, which entered into force on 1 January 2020. In 2017, the Chinese Ministry of Ecology and the Environment included microplastics in cosmetics and cleaning products on the list of high-risk environmental products. More information about these restrictions was presented by China’s National Development and Reform Commission (NDRC) in notice No.80 called “Opinions on Further Strengthening the Control of Plastic Pollution”, which was published on 16 January 2020. This notice aims to ban the use of microbeads in personal care and cosmetic products [122]. Later, published notice No.1146 of 10 July 2020 defines products where plastic microbeads smaller than 5 mm are banned. It includes rinse-off products such as shampoo, face cleaners, and toothpastes. However, this report also includes information about the ban of ultra-thin plastic bags, non-degradable plastic bags, plastic straws, non-degradable plastic tableware, non-degradable plastic packing bags, non-degradable plastic tapes, etc., by the end of 2025 in almost every prefecture [123].

According to Chemical Watch sheets and NDRC notices, this restriction is expected to start on 31 December 2020, when these products will no longer be manufactured. Subsequently, the deadline for the sale of inventory will run and a total ban on the sale of these products will occur on 31.12.2022 [122,123,124]. It can be expected that these restrictions will significantly affect the amount of targeted microplastics, as China occupies an essential position in the cosmetics market.

### 5.4. Legislation in the World

Argentina is the first state in South America that banned the manufacturing, import, and sale of cosmetic products with intentionally added microplastic particles. Law 27602 was ratified on 30 November 2020 and published on 29 December 2020 [125]. Since 2018, the UK has enforced the so-called “Environmental Protection (Microbeads) (England) Regulations 2017” with attached regulations for Wales and Scotland, which banned rinse-off personal care products containing plastic microbeads and their manufacture and sale. This regulation was marked as one of the strictest acts in the field of microplastics [126]. Canada has their “Microbeads in Toiletries Regulations SOR/2017-111”. Toiletries means products for hair, skin, teeth and mouth care, which contain plastic microbeads. The manufacturing, import, and sale of these products were prohibited since 1 July 2018 (1 July 2019 for stock sales) [127]. Legislation in other countries that ban some types or sources of microplastics is likely similar. The prohibition of microbeads in rinse-off cosmetic products has been in force or in the legislation process in South Korea, Australia, India, and Taiwan [128].

## 6. Conclusions

Microplastics have been found in all environments and undoubtedly will be a problem in the future. Countries in Europe and beyond seek preventive measures to address this problem. The occurrence, detection, and characterization of microplastics in the aquatic environment has been the subject of a large number of studies. The current state of the identification and evaluation of microplastics in the aquatic environment is relatively heterogeneous; published detection methods vary and are not easily replicable. Many studies lack clear information on sampling, sample processing, laboratory preparation, and the methodology of the polymer identification itself. These disproportions in the methodology are evident from Table 1, which includes an overview of the published methods for the determination of microplastics from the environment. All this reduces the reproducibility and comparability of the results. It is thus important to focus even more strongly on the determination of microplastics in the aquatic environment, and to develop a high-quality methodology for their evaluation in water in general.

The call for the importance of agreeing on a unified methodology builds, inter alia, on our summary of 30 examples (Table 1). In many cases, results were presented in so many different ways, which makes it impossible to compare data between studies. Moreover, the methods of sampling and sample preparation even differed in studies with the same types of samples. Overall, there is a low number of studies that would replicate methodologies from previous studies, which we claim is a way to improve data replicability, comparability and thus their reliability. At a time of increasing production and consumption of plastic material, it is necessary to work on the creation of a unified methodology for the determination of microplastics because microplastics are subsequently fragmented into nanoplastics, which can have probably a greater impact on human health and the quality of the aquatic environment. It is likely that this problem causes difficulties in the preparation of new legislation and its subsequent control. We appreciate the new legislation documents, but due to the transport of microplastic particles over vast distances, it is necessary to introduce laws restricting the production and formation of microplastics all around the world. We also highly recommend preparing waste management legislation, which can significantly reduce the amount of plastic waste entering the aquatic environment. Equally important is the responsible behavior of people, which can be achieved through quality educational campaigns run by world organizations.

Findings:

- Legislation in the world focuses mainly on restrictions in the field of primary microplastics and disposable plastic products.
- Results presented in studies about microplastics in the aquatic environment are often incomparable.
- The descriptions of the same methods in studies often differ, which causes problems with replicability.
- Unification of the methodology for the detection of microplastics in the environment is the only way to trace back the effectiveness of restrictions.

## Figures and Tables

**Table 1:** Information about selected studies of microplastics in fresh water and groundwater, sea water, and sediments.

Author	Sampling Location	Sampling Method	Sample Volume	Pre-Treatment	Instrumental Analysis Method	Amount of Particles	Ref.
**Fresh Water and Groundwater**							
Bharath K et al. (2021)	Chennai Metropolis, South India	Silanized amber glass bottles with Teflon-lined caps	1 L each (20 samples total)	Filtration (0.45 mm Whatman cellulose nitrate filter paper)	Dissecting microscope, ATR-FTIR (JASCO 6600 Type-A, Easton, USA)	3–23 MPs per L	[75]
Selvam et al. (2021)	Tamil Nadu, South India	Water pump (2 V DC Teflon pump with stainless-steel sieve; 50 μm)	approx. 2–4 L each (24 groundwater samples + 20 fresh water samples)	Chemical digestion (30% H_2_O_2;_ Fe (II) solution), filtration (micro-line filter paper)	Stereomicroscope (XRZ3, Olympus), ATR-FTIR	groundwater: median of 4.2 MPs per L maximum of 10.1 MPs per L fresh water: median of 7.8 MPs per L maximum of 19.9 MPs per L	[76]
Quesadas-Rojas et al. (2021)	Rio Lagartos, Mexico	Zooplankton net (200 μm)	6.76 × 10^5^ m^3^	Wet sieving (5 mm; 0.3 mm), chemical digestion (diluted HCl), wet peroxidation (hydrogen peroxide and FeSO4)	Stereomicroscope (Leika EZ4, 35×)	median of 21.30 MPs per kg * maximum of 328.10 MPs per kg * * of dry matter	[77]
Liu et al. (2021)	Rivers and lakes—Tibetan Plateau	Water collector (N/A)	N/A	Density separation (CHKO_2_ in deionized water—1.5 g/cm^3^), filtration (GF/C filters)	Raman microscope spectroscopy	247–2686 MPs per m^3^ average: 856 MPs per m^3^	[78]
Yan et al. (2021)	Qinhuai River, China	Water pump	20 L	Chemical digestion (Fenton’s reagent), vacuum filtration (5 μm, GF/A, Whatman)	Stereomicroscope (Olympus SZX10)	1467–20,567 MPs per m^3^	[79]
He et al. (2021)	Yangtze River, China	Trawl (300 μm)	50 mL	Sieving (5 mm; 2.8 mm; 1 mm; 300 μm), visual analyzing, chemical digestion (30% H_2_O_2_), density separation (NaCl), vacuum filtration (0.7 μm GF/F)	Stereomicroscope (M165FC, Leica, Wetzlar Germany); FT-IR ATR (Vertex 70, Bruker, Germany)	from 1.62 ± 0.6 × 10^5^ to 4.25 ± 3.87 × 10^6^ MPs per km^2^	[80]
He et al. (2021)	Yangtze River, China	Water pump	40 L each	Sieving (5 mm; 2.8 mm; 1 mm; 300 μm), visual analyzing, chemical digestion (30% H_2_O_2_), density separation (NaCl), vacuum filtration (0.7 μm GF/F)	Stereomicroscope (M165FC, Leica, Germany), FTIR ATR	from 800.0 ± 300.0 to 3088.9 ± 330.6 MP/m^3^	[80]
Huang et al. (2021)	West River, China	Stainless steel drum	30 L each	Filtration (75 μm), chemical digestion (30% H_2_O_2_), vacuum filtration (0.45 μm GF/F)	Metallographic microscope (MV5000(R/TR)), FTIR	2.99–9.87 MPs per L	[81]
Suteja et al. (2021)	Benoa Bay, Bali	Mini manta trawl (300 μm)	N/A	Separation (tweezers), filtration (5 mm; 200 μm), chemical digestion (30% H_2_O_2_), vacuum filtration (0.45 μm GF/F)	Stereomicroscope (Nikon Eclipse Ni-U), FTIR (JASCO FTIR Microscopes IRT-7200 VC)	average: 0.62 MPs per m^3^ maximum: 1.41–1.88 MPs per m^3^	[82]
Napper et al. (2021)	Ganges River, South Asia	Water pump	30 L each	N/A	Light microscope (S9E-Leica), FTIR with a Hyperion 1000 microscope coupled to a Vertex 70 spectrometer (Bruker)	average: 0.038 ± 0.004 MPs per L	[83]
**Sea Water**							
Russell and Webster (2021)	Scotland	Neuston net (335 µm)	from 16 to 557 m^3^	Sieving	Micro-FTIR	4565 MPs per km^−2^	[84]
Zhou et al. (2021)	Maowei Sea	Steel bucket	5 L each	Filtration (nylon membrane 5 µm pore size), chemical digestion (10% KOH)	Stereomicroscope, micro-FTIR	1.47–7.61 MPs per L	[85]
Manbohi et al. (2021)	Caspian Sea	Plankton net (300 µm)	141.37 m^3^	Sieving (5 mm), filtration (S&S filter papers, <2 µm mesh size)	Stereomicroscope, polarized light microscope, FE-SEM with EDS, micro-Raman spectroscope	0.246 ± 0.020 MPs per m^3^	[86]
Jiang et al. (2020)	Nordic Seas	Water pump	100 L each	Filtration (stainless-steel mesh—5 mm, plankton net—50 µm), chemical digestion (30% H_2_O_2_), density separation (ZnCl_2_—1.6 g/mL), sieving (stainless steel mesh—2, 1, 0.5, 0.1, 0.05 mm)	Stereomicroscope, FTIR (only randomly selected MPs ground with potassium bromide, n = 200), scanning electron microscopy (SEM; randomly selected, n = 24), X-ray spectroscopy (EDS)	from 2.43 ± 0.84 to 1.19 ± 0.28 MPs per L	[87]
Berov and Klayn (2020)	Black Sea	Manta net (300 µm)	84.5 ± 6.3 m^3^	Chemical digestion (3% H_2_O_2_—only hand sorted particles)	Stereomicroscope, optical microscope	4.62 × 10^4^ MPs per km^−2^, 0.62 MPs per m^−3^	[88]
Jiang et al. (2020)	South Yellow Sea	Water pump	100 L each	Filtration (stainless-steel mesh—5 mm, plankton net—50 µm), chemical digestion (30% H_2_O_2_), density separation (ZnCl_2_—1.6 g/mL), sieving (stainless steel mesh—2, 1, 0.5, 0.1, 0.05 mm)	Stereomicroscope, hot needle test, FTIR (only randomly selected items)	from 4.5 ± 1.8 to 6.5 ± 2.1 MPs per L	[89]
Taha et al. (2021)	South China Sea	Water pump	2.041 L per second for ~10 min	Filtration (net—20 μm; glass-fiber filters—0.4 μm)	Stereomicroscope, scanning electron microscope (SEM), micro-FTIR-ATR	1687 MPs per m−3 (estuary); 1900 MPs per m−C15:G203 (offshore)	[90]
Tošić et al. (2020)	White Sea; Barents Sea; Kara Sea	Manta trawl (330 µm)	N/A	Sieving (5;1;0.3 mm mesh size)	FTIR, Nile Red staining	from 28,000 to 963,000 MPs per km^2^	[91]
Hosseini et al. (2020)	Oman Sea	Water pump	10 L (each site; only 100 mL per sample was used)	Chemical digestion (30% H_2_O_2_), filtration (glass microfiber filter; 1.2 mm)	Light microscope, micro-FTIR (only randomly selected particles; n = 150)	218 ± 17 MPs per L	[92]
Zhang et al. (2020)	Bohai Sea	Manta net (330 µm); Water pump	50 L (Water pump); N/A (Manta net)	Wet sieving (5 mm; 0.3 mm), chemical digestion (0.05 M Fe II; 30% H_2_O_2_), density separation, filtration (glass-fiber filters; 0.7 µm)	Stereomicroscope, micro-FTIR	0.35 ± 0.13 MPs per m^3^	[93]
**Sediment**							
Quesadas-Rojas et al. (2021)	Rio Lagartos in Mexico	Ponar dredge	2.2 L	Density separation (CaCl_2_—1.4 g/mL), wet sieving (5 mm and 0.3 mm), chemical digestion (diluted HCl), wet peroxidation (hydrogen peroxide and FeSO_4_)	Stereomicroscope (Leika EZ4, 35×)	median of 21.30 MPs/kg * maximum of 328.10 MPs/kg * * of dry matter	[77]
Liu et al. (2021)	Rivers and lakes—Tibetan Plateau	Sieving (1 mm)	N/A	Density separation (CHKO_2_ + in deionized water—1.5 g/cm^3^), filtration (GF/C filters)	Raman microscope spectroscopy	0–933 MPs per m^2^ average: 362 MPs per m^2^	[78]
Yan et al. (2021)	Qinhuai River, China	Peterson grab sampler	1 kg	Density separation (NaCl—1.2 g/cm^3^), stirring (10 min.) and settling (4 h), sieving (54 μm filter), flotation (ZnCl_2_—1.5 g/cm^3^)	Stereomicroscope (Olympus SZX10)	1115–6380 MPs per kg	[79]
Huang et al. (2021)	West River, China	Rab bucket (B-10104, Ravenep)	5 kg	Density separation (NaCl), chemical digestion (30% H_2_O_2_), 100 rmp thermostatic oscillation (60 °C)	Metallographic microscope (MV5000(R/TR)), FTIR	2560–10,240 MPs per kg	[81]
Kreitsberg et al. (2021)	Baltic Sea, Estonia	By hand using scuba equipment	1 kg	Density separation (NaCl), stirring, vacuum filtration (GF/D, 2.7 μm)	Stereomicroscope (Leica M165 FC), FTIR	0–1817 MPs per kg median: 208 MPs per kg	[94]
Xia et al. (2021)	Liangfeng River, China	Stainless steel shovel	20 g (dry weight)	Density separation (NaCl—1.2 g/mL), filtration (stainless sieve; 50 μm), chemical digestion (30% H_2_O_2_), filtration (polycarbonate filter membrane; 10 μm)	Nile Red staining, laser confocal microscope (Revolution XD, Andorra, UK), imaging under blue light, particle counting (ImageJ), stereomicroscope (XTL-165-LT, Phmias, China), FTIR (Nicolet iS10, PE, USA)	Rainy season: 33,200 (±11,990) MPs per kg; Dry season: 27,900 (±15,050) MPs per kg	[95]
Chinfak et al. (2021)	Bandon Bay, Thailand	Ekman grab sampler	300 g (wet sediment)	Density separation (NaCl—1.2 g/cm^3^), filtration (nylon membrane filter; 5 μm),	Stereomicroscope (LEICA MZ9.5), hot needle test, FTIR (randomly selected; n = 126)	from 5 to 160 MPs per kg (dry weight)	[96]
Radhakrishnan et al. (2021)	Kayamkulam Estuary, India	Van Veen grab sampler	30 g (for analysis)	Wet sieving (5 mm), chemical digestion (30% H_2_O_2_, 2 N HCl), density separation (ZnCl_2_—1.58 g/mL), filtration (nitrocellulose membrane filter; 0.45 μm)	Stereomicroscope, FTIR-ATR, SEM	438.8 and 421.5 MPs per kg (left and right arm of the estuary)	[97]
Kiss et al. (2021)	Tisza River, Central Europe	Iron spatula	1 kg (wet sediment)	Sieving, density separation (ZnCl_2_—1.8 g/mL), chemical digestion (30% H_2_O_2_)	Visual identification, digital microscope (Ash Inspex3 HD)	3177 ± 1970 MPs per kg (Tisza River); 3808 ± 1605 MPs per kg (tributaries)	[98]
Felismino et al. (2021)	Simcoe Lake, Canada	Petite Ponar	from 11.2 to 120.2 g (dry weight)	Sieving (45 µm), density separation (1.4 g/mL CaCl_2_)	OMANO microscope, visual identification and sorting, Raman spectroscopy, FTIR-ATR	372 ± 346 MPs per kg (dry weight)	[99]

FTIR (infrared spectroscopy with Fourier transformation); ATR (attenuated total reflection); SEM (scanning electron microscopy); EDS (energy dispersive X-ray spectroscopy).

**Table 2:** Instrumental analysis methods used in studies from Table 1 (data from Table 1).

Instrumental Analysis Method	Number of Studies
FTIR	21
Raman spectroscopy	4
SEM	4
EDS	2
